# Molecular Chaperone Dysfunction in Neurodegenerative Diseases and Effects of Curcumin

**DOI:** 10.1155/2014/495091

**Published:** 2014-10-19

**Authors:** Panchanan Maiti, Jayeeta Manna, Shobi Veleri, Sally Frautschy

**Affiliations:** ^1^Department of Neurology, University of Tennessee Health Science Center, Memphis, TN 38163, USA; ^2^Department of Physiology, University of Tennessee Health Science Center, Memphis, TN 38163, USA; ^3^Neurobiology-Neurodegeneration and Repair Laboratory, National Eye Institute, National Institutes of Health, Bethesda, MD 20892, USA; ^4^Veteran's Greater Los Angeles Healthcare System, Geriatric Research and Educational Core, Los Angeles, CA 90073, USA; ^5^Departments of Neurology and Medicine, University of California at Los Angeles, Los Angeles, CA 90095, USA

## Abstract

The intra- and extracellular accumulation of misfolded and aggregated amyloid proteins is a common feature in several neurodegenerative diseases, which is thought to play a major role in disease severity and progression. The principal machineries maintaining proteostasis are the ubiquitin proteasomal and lysosomal autophagy systems, where heat shock proteins play a crucial role. Many protein aggregates are degraded by the lysosomes, depending on aggregate size, peptide sequence, and degree of misfolding, while others are selectively tagged for removal by heat shock proteins and degraded by either the proteasome or phagosomes. These systems are compromised in different neurodegenerative diseases. Therefore, developing novel targets and classes of therapeutic drugs, which can reduce aggregates and maintain proteostasis in the brains of neurodegenerative models, is vital. Natural products that can modulate heat shock proteins/proteosomal pathway are considered promising for treating neurodegenerative diseases. Here we discuss the current knowledge on the role of HSPs in protein misfolding diseases and knowledge gained from animal models of Alzheimer's disease, tauopathies, and Huntington's diseases. Further, we discuss the emerging treatment regimens for these diseases using natural products, like curcumin, which can augment expression or function of heat shock proteins in the cell.

## 1. Introduction

Current knowledge in the field supports the idea that accumulation of misfolded or mutant proteins inside and or outside neurons is an early event contributing to neurodegenerative disorders [1, 2]. Neuron dysfunction caused by abnormal protein aggregation represents a major and unresolved medical challenge. The clinical manifestations depend on the afflicted brain region and may involve disruption of daily activities including sensory and motor functions such as moving, speaking, swallowing, breathing, or cognitive dysfunction. In particular, misfolding and aggregation of proteins are thought to be a major cause of synaptic loss and neuronal death observed in different neurodegenerative diseases [1, 3]. Removal of aggregates can occur via different cellular processes, which, depending on the milieu, may aggravate or attenuate the disease process associated with Alzheimer's, Parkinson's, Huntington's, prion diseases, frontotemporal dementia, and motor neuron disease. Most importantly, to prevent the toxicity associated with misfolded protein aggregates, early diagnosis is critical to facilitate treatment prior to significant loss of neurons and accumulation of misfolded proteins and the onset of clinical symptoms. The mechanisms involved to clear protein aggregates in the cell are several, including molecular chaperones, the ubiquitin proteasome system, and autophagy pathways [4, 5]. Although a moderate degree of autophagy has been shown to play a role in life extension, overstimulation of autophagy including macroautophagy can lead to cell death [6]. Therefore, molecular chaperone-mediated autophagy is thought to be the most specific and promising therapeutic approach to remove protein aggregates in the cells for neurodegenerative diseases [4, 5, 7, 8]. Therefore, not surprisingly, there has been an escalation in research on role of molecular chaperones in degradation of misfolded protein debris in different neurodegenerative diseases [2, 9].

Molecular chaperones, such as heat shock proteins (HSPs), are part of a highly conserved cellular defense system, which regulates various cellular functions [10, 11]. They provide protection against deleterious cellular stress by interaction with different cochaperones and other partner-proteins or by inducing expression of kinases, hormone receptors, transcription factors, or antioncogenic proteins [11, 12]. The principal roles of HSPs are to fold nascent polypeptides to their appropriate conformation, refold mild denatured/damaged proteins, prevent protein aggregation, and degrade severely damaged proteins and apoptosis [13–17]. The HSPs help to degrade the proteins by delivering them to the ubiquitin proteasome system [10]. They cannot complete these complex tasks alone, rather they require cochaperones and many other client-proteins such as kinases [18]. Although most of the cellular proteins can fold independently, HSPs are essential to efficiently facilitate the protein folding process [19]. Therefore, failure of these essential cellular quality control mechanisms leads to pathogenic conditions, as seen in different neurodegenerative diseases [20].

## 2. Protein Misfolding and Aggregation Cause Neuronal Dysfunction

Gradual accumulation of denatured or misfolded proteins over time leads to progressive loss of structure and/or function of neurons and ultimately neuronal death [3, 21]. In most cases, the abnormal proteins are eventually deposited as insoluble intracellular or extracellular aggregates as amyloid plaques or intracellularly, for example, in neurofibrillary tangles, conditions known as amyloidosis, a process associated with neurodegeneration [3] (Table 1). The aggregation of misfolded proteins is highly regulated by both genetic and environmental factors [22].

**Figure 1:**
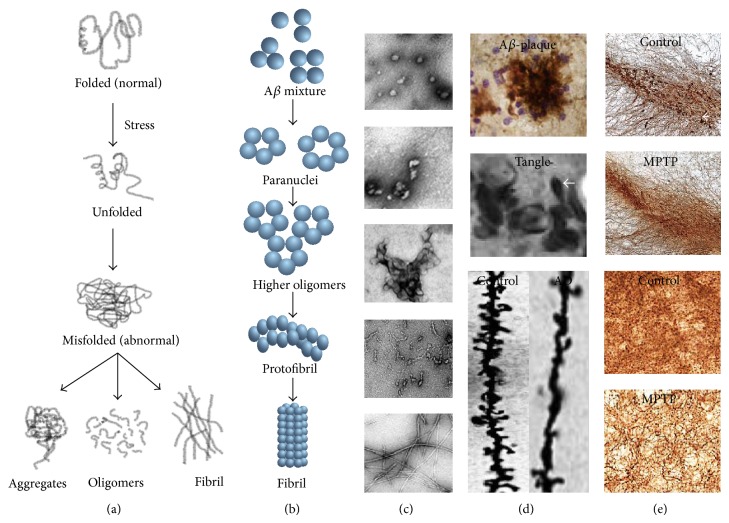
Protein misfolding and aggregation in different neurodegenerative diseases. (a) Schematic diagram showing steps of formation of different abnormal protein species after the cellular stress response; (b) schematic diagram of sequential formation of different amyloid species; (c) electron micrographs of different species of amyloid beta protein; (d) amyloid beta plaques (upper) stained with 4G8 antibody from brain tissue of AD mouse model, neurofibrillary tangle (middle), and synaptic (dendritic spine, Golgi-Cox stain) loss in mouse model of Alzheimer's disease (lower); (e) tyrosine hydroxylase (the indicator of dopaminergic neuron) positive (small arrow) neuronal loss in the substantia nigra pars compacta and tyrosine hydroxylase positive fibre loss in the striatum of MPTP-model of Parkinson's disease.

Interestingly, most of the amyloid proteins (e.g., amyloid-*β*, tau, *α*-synuclein, huntingtin, prion, etc.) are metastable and noncrystalline in nature and have similar native structures [23, 24]. Normally when proteins undergo conformational change in specific conditions and become denatured or misfolded, they are refolded in endoplasmic reticulum (ER) or degraded in the lysosome, but failure of this system leads to accumulation of insoluble amyloid aggregates (Figure 1). The current thought is that soluble, prefibrillar protein aggregates, particularly dodecamers (or larger oligomers) with specific conformations, are more neurotoxic than deposited amyloid fibrils [25]. Direct therapeutic targeting of aggregates is challenging because (1) the prefibrillar protein aggregates can have multiple structural conformations, (2) not all aggregates will be toxic, and (3) the different toxic species may be toxic through different mechanisms.

## 3. Prevention of Neurodegeneration: Target Is Undefined

Although the neurophysiological mechanisms of protein misfolding diseases are still not clear. Researchers are beginning to tackle the problems of aggregated proteins by applying a multifaceted approach to stop further neuronal death. Prior directions in drug development for AD have yielded disappointing results in trials, including strategies to (1) decrease the production of amyloidogenic proteins by inhibiting the rate-limiting enzyme responsible for production of the amyloidogenic protein (e.g., inhibition of *β* and *γ* secretases that produce A*β* in AD), (2) directly inhibit aggregation, and (3) promote phagocytic clearance of oligomers or amyloid by passive or active immunization or 4-inhibit enzymes that phosphorylate tau. Nevertheless it is untested whether combination therapies of these strategies will be effective. Further there is increased attention to increase neuroprotection by restoring endogenous pathways for maintaining protein homeostasis to enable effective protein degradation pathways and protein refolding, the cellular defense mechanisms against proteinopathies. There has recently been a focus on small molecules/drugs, bioactive phytochemicals, which can impact proteostasis and also exert neuroprotection through antioxidant and anti-inflammatory activity, particularly molecules classified by the Food and Drug Administration as generally recognized as safe (GRAS).

## 4. Boosting the Cellular Defense System Can Protect against Proteinopathies

Research efforts to boost the cellular defense system to treat neurodegenerative disease are underway. Unlike in disease, normally HSPs maintain protein quality control by several mechanisms including folding nascent protein, refolding and reactivation of unfolded and misfolded proteins, assembly and disassembly of macromolecular protein structures, and targeting abnormal and inactive proteins for degradation [5, 26, 27]. Different HSPs can be transported to synapses and axons and prevent aggregation of misfolded proteins. Recent experimental evidence suggests that HSPs have a significant role in direct inhibition of aggregation of those amyloidogenic proteins such as A*β*, tau, HTT, and *α*-synuclein and also promote ubiquitination and degradation of aggregated or misfolded proteins [28]. HSPs reduce the formation of annular, perhaps pore-forming species in favor of amorphous structures and fibrils as shown for the HTT fragment, suggesting prevention of channel formation [4]. In particular, the HSPs especially HSP40 and HSP70 regulate the disposal of toxic tau, A*β*, *α*-synuclein, and HTT aggregates via multiple mechanisms [5, 26, 27]. HSPs may bind* in vitro* and/or* in vivo* to prefibrillar mutant HTT [4], hyperphosphorylated tau, and *α*-synuclein aggregates [3] to interfere with the formation of oligomer or higher order structures and to regulate the ubiquitin proteasome and the autophagic-lysosomal pathways [29]. Further, elevated levels of HSPs have been observed in activated astrocytes to target those proteins for degradation [8]. Besides protein refolding or degradation, HSPs also assist in a specialized autophagy mechanism called chaperone-mediated autophagy (CMA). This is a highly selective and constitutive subtype of autophagy that utilizes chaperone proteins and lysosomal receptors to directly target proteins that contain a consensus pentapeptide motif, for example, KFERQ, and translocate them into the lysosomal lumen for their degradation [30]. In this case, the lysosome associated heat shock cognate 70 (Hsc70, a constitutively expressed chaperone) binds with target protein to form a substrate-chaperone complex and then is transported to the surface of endoplasmic reticulum where it can specifically bind with lysosomal receptor protein LAMP-2A. This is followed by unfolding, multimerization of LAMP-2A, and finally translocation of the target protein into lumen of ER for their final degradation. The interaction of LAMP-2A with Hsc70-protein complex requires its stable structure and HSP90 to maintain this structure [31]. However, the failure of this important mechanism has been linked to the pathogenesis of several major neurodegenerative diseases [26, 32]. The crucial roles of HSPs in different neurodegenerative disease are discussed further.

## 5. Characterization of Heat Shock Proteins and Their Functions

In general, most eukaryotic cells constitutively express many chaperones and cochaperones, which are further modified by cellular stress. They are classified according to approximate molecular size or function into six conserved classes: HSP40, HSP60, HSP70, HSP90, HSP100, and the small HSPs (15 to 30 kDa, HSP27, HSP10) [33] (Table 2). They are localized in the nucleus, cytoplasm, and cellular organelles, such as mitochondria and ER [33]. Most importantly, HSPs are tightly regulated requiring a precise balance, while overproduction of certain HSPs can lead to diseases including cancer [12]. However, among all HSPs, low molecular weights (MW) HSPs are ATP-independent for their chaperone activity, whereas high MW HSPs are ATP-dependent [13, 16, 17].

*Small HSPs (sHSPs).* The small HSPs are low MW chaperones ranging from 12–43 kDa [34], localized in the cytosol as well as in different subcellular compartment including the ER and nucleus. Their expression varies with cellular environment and also in different neurological disorders. The most abundant and well understood sHSP is HSP27, while less is known about the less abundant sHSPs such as HSP10, HSP12, HSP20, and HSP26. Almost all these sHSPs share some common features including a small MW ranging from 12 to 43 kDa, a conserved *α*-crystalline domain of ~90 residues, the ability to form a large oligomers, and a dynamic quaternary structure. They have high chaperone activity that can attenuate protein aggregation, and increased expression is predominantly induced by cellular stress [34, 35]. In addition to chaperone activity, the sHSPs are also involved in thermotolerance, cell development and differentiation, signal transduction, and inhibition of apoptotic cell death* in vivo*. They also serve as a cochaperone of HSP70 and assist inhibition of protein aggregation [34]. Other important functions of sHSPs are to assist in ubiquitin proteasome degradation of misfolded or denatured proteins. Further, they can increase glutathione to protect cells from oxidative damage. Finally, HSPs also have the ability to interact with actin and intermediate filaments and prevent their damage [35].

**Figure 2:**
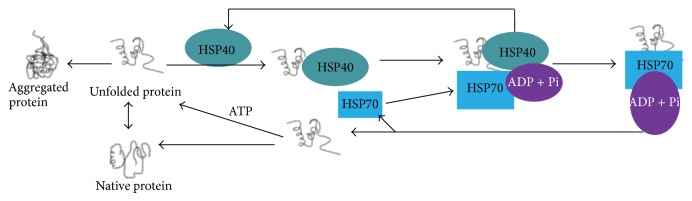
Role of HSP40 in protein folding and degradation. HSP40 acts as cochaperones of HSP70 and maintains ATPase activity required for proper function of HSP70.

*HSP40.* HSP40 also known as chaperone DnaJ is a large protein family, which are the main cochaperones for HSP70 [36]. However HSP40 can also bind to aggregates and limit further aggregation or refold them without HSP70. There is little known about dysregulation of HSP40 in neurodegenerative disease; but it colocalizes with deposits and HSP40, and other HSPs correlate negatively with tau oligomers, an indicator of severity of disease [37]. Data using neurodegenerative cellular models of prion [38] or polyglutamine [39] aggregation argue a beneficial effect on limiting aggregation. It is expressed in a variety of organisms with different isoforms typically with three isoforms. All types of HSP40 contain a highly conserved J domain, which interact with HSP70 ATPase domain. Thus, HSP40 regulates ATPase activity of HSP70 (Figure 2). Further, it can carry substrate with an appropriate conformation to HSP70. Therefore, by stimulating the ATPase activity of HSP70, HSP40 is primarily associated with unfolded polypeptide chains and can reduce aggregates by protein translation, folding, unfolding, translocation, or degradation [40].

*HSP60.* HSP60 is a heptameric 60 kDa mitochondrial chaperone. HSP60 works together with HSP70 for protein folding. Furthermore, it plays key roles in mitochondrial protein transport, replication, and transmission of mitochondrial DNA and apoptosis. For actin and tubulin, HSP60 is a specific chaperone. The main function of HSP60 is to assist protein folding and maintain the structural conformation [41, 42]. In addition, HSP60 transports and maintains mitochondrial proteins required for its DNA replication. Finally, cytoplasmic HSP60 interacts with antiapoptotic proteins to prevent cell death [43]. Depending on the demand and cellular milieu, HSP60 moves to and from the cytosol and mitochondria [41–43].

*HSP70.* The most conserved chaperone in all living organisms is HSP70 (MW 70 kDa), which is localized in cytosol and also in organelles such as the endoplasmic reticulum (ER). HSP70, which makes complexes with unfolded or partially denatured proteins, has two functional domains: the ATPase domain and the substrate-binding domain (SBD). The activities of these domains are controlled by the availability of cellular ATP level. The ATPase domain of HSP70 supplies the required energy for the protein folding and maturation. Similarly, HSP70 binding to misfolded peptides increases the ATP hydrolysis. Further, HSP70 can interact with HSP40 and HSP90 to perform the process of protein folding. There is abundant evidence supporting a neuroprotective role in protein misfolding diseases including all of neurodegenerative disorders [44–46].

**Figure 3:**
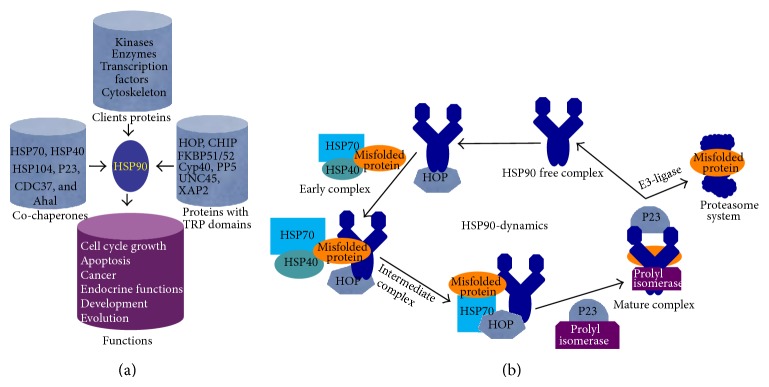
Schematic diagram of role of HSP90 and its cochaperones and client proteins in protein folding and degradation. (a) With the help of cochaperones, clients, and proteins with a TRP domain, HSP90 maintains protein quality control, cell cycle growth, development, and other essential activities of the cell. (b) Structural changes of HSP90 during protein folding and degradation. HSP90 dynamically changes from “open” to close structures. In its open form, it can bind with misfolded proteins along with other chaperones (early complex) and become a “closed” structure (mature complex). After releasing misfolded protein for proteasomal degradation, it goes to an “open” form (HSP90 free complex).

*HSP90.* The most abundant molecular chaperone in the cell is HSP90, a 90 kDa MW dimeric protein. It is one of the main HSPs, playing an important role in protein misfolding that stabilizes and activates more than 200 HSP90-client proteins required for cell signaling and adaptive responses to stress [47–49]. It is mainly localized in the cytosol but sometimes in the ER and mitochondria. HSP90 is a dimeric protein, which has a highly conserved N-terminal domain and a C-terminal domain (Figure 3). In the human proteome, inducible (HSP90*α*) and constitutive (HSP90*β*) forms of HSP90 have been identified. The HSP90 is one of the main cytosolic molecular chaperones, which is activated with HSP40 and HSP70 [4, 5, 8] (Figure 3(b)). The main function of HSP90 is that it can stabilize certain protein complexes and assist in protein degradation (Figure 3(b)). However, its overexpression has been observed in cancers [26, 49]. For HSP90 to acquire the full active molecular chaperone activity HSP90, it requires a series of functionally related cochaperones, client/partner proteins, and multimeric protein complexes, like HOP, CDC37, P23 and Aha1, immunophilins (FKBP51 and FKBP52), peptidyl-prolyl isomerases and cyclophilin CYP40, and so forth [4, 50] (Figure 3(a)).

**Figure 4:**
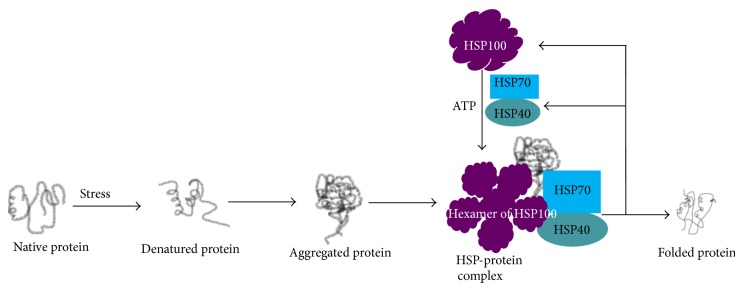
Role of HSP100 in protein folding and degradation. Along with HSP40 and HSP70, the hexameric form of HSP100 binds to the aggregated/misfolded protein to correct folding.

*HSP100/104.* The HSP100 is a hexameric cytosolic protein with great diversity of functions especially high thermotolerance [51]. Its MW ranges from 100 to 110 kDa, and originally discovered as ClpA, ClpB, and ClpC subfamilies [52]. In normal growth conditions the cells do not require HSP104/110, but its expression may be induced under extreme heat or exposure to other harsh environments to protect the cell and promote proteolytic degradation of specific cellular protein debris. In addition, sometimes HSP104/110 expression is required for regulation of transcription. Like HSP70, it has two main domains, the ATP binding region and the substrate-binding domain. HSP104/110 is a subclass of molecular chaperones that has the ability to solubilize almost any protein that becomes aggregated after severe stress [51] (Figure 4). Further, with the help of other chaperones such as HSP70 and HSP40, HSP100 breaks large protein aggregates into smaller aggregates and thus facilitates their proteasomal degradation [51]. Finally, HSP100 acts as a remodeling machinery, attempting to refold a misfolded protein or participate in the clearance of irreversible protein aggregates [51] (Figure 4).

## 6. Role of HSPs in Different Neurodegenerative Diseases

**Figure 5:**
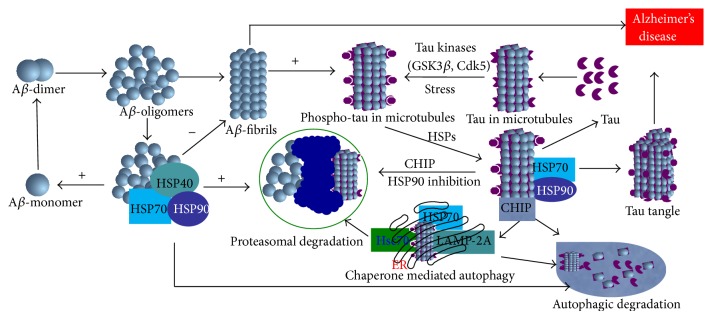
Role of molecular chaperones in Alzheimer's disease. HSP90, HSP70, and other client proteins are directly involved in degradation of A*β* oligomers and phosphorylated tau containing microtubule and help them to degrade either proteasome pathway or through autophagy pathway. Failure to dephosphorylate by HSPs leads to formation of tau tangle, a hallmark of AD.

*Alzheimer's Disease.* The principal misfolded proteins in Alzheimer's disease (AD) are A*β* and tau [1]. The first one is formed from amyloid precursor protein (APP) and deposited as amyloid plaques, mostly in extracellular spaces. Tau is the microtubule stabilizing protein and when it is hyperphosphorylated accumulates intracellularly (and sometimes extracellularly) in neurofibrillary tangles [26, 32, 53, 54]. However, HSPs especially HSP70 can bind with APP and interfere with the APP secretory pathway to reduce the production of both A*β*40 and A*β*42 [55]. Dickey et al. reported that HSP70 and HSP90 interact with tau and A*β* oligomers and degrade them through proteasome system [26] (Figure 5). Similarly, overexpression of HSP70 decreases the amount of insoluble tau, reduces tau phosphorylation, increases tau stability, promotes tau binding to microtubules, and decreases the* in vitro* and* in vivo* toxicity associated with tau protein [56] (Figure 5). In contrast, downregulation of HSPs by RNA-mediated interference (RNAi) has the opposite effect [5]. Heat shock cognate 70 (HSC70) together with HSP70 or HSP90 can directly bind to tau, independent of its phosphorylation status, thus facilitating microtubule polymerization and limiting tau aggregation [57]. In addition, HSP90 and its dependent cochaperones and client proteins may be essential for refolding denatured or misfolded tau and A*β* [57]. Both HSP70 and HSP90 can promote tau solubility and tau binding to microtubules as well as reduce insoluble tau and tau phosphorylation [5]. Overexpression of inducible HSP70 reduced soluble and insoluble tau levels in 30-month-old mice [5, 58]. Levels of HSP90 are inversely associated with granular tau oligomers and neurofibrillary tangles in AD [37] and in a mutant tau model [5] (Figure 5). In addition to facilitating the removal of aggregates by chaperone-mediated autophagy (CMA), HSPs are known to prevent caspase activation, for which substrates include tau, APP, and HTT [59].

**Figure 6:**
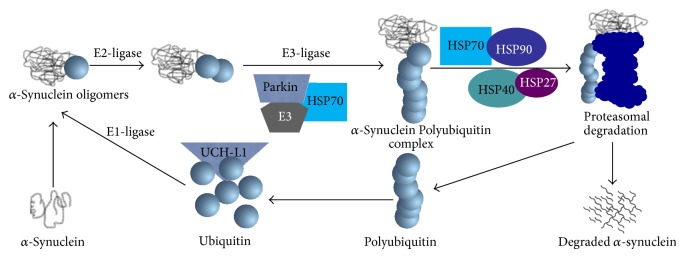
Role of different HSPs in degradation of *α*-synuclein in Parkinson's disease. HSP90, HSP70, HSP40, and HSP27 bind with polyubiquitinated *α*-synuclein for proteasomal degradation to prevent dopaminergic neuronal loss in the substantia nigra.

*Parkinson's Disease.* Parkinson's disease (PD) is characterized by gradual debilitation due to selective degeneration of dopaminergic neurons in the substantia nigra pars compacta (SNpc) with a subsequent decline in dopamine (DA) in the nigrostriatal pathway [60–65]. Most PD cases are sporadic, and in rare cases it may be inherited. The common symptomatic feature of PD is bradykinesia, tremor, rigidity abnormalities in gait, and posture. The hallmark pathology of PD is accumulation of *α*-synuclein, the main component of Lewy bodies in midbrain dopaminergic neurons [60–65]. Examination of Lewy bodies revealed the presence of not only *α*-synuclein, but a variety of other proteins including, neurofilaments, ubiquitinated proteins, and several HSPs (HSP70 and HSP90) [66]. HSP70 is localized with *α*-synuclein, dopamine transporter (DAT), parkin, proteasome subunits, and ubiquitin with ubiquitin carboxy-terminal hydrolase-L1 (UCH-L1) [67]. HSP70 can prevent dopaminergic degeneration in PD due to its antiapoptotic activities [68, 69]. Further, HSP70 can enhance parkin binding and ubiquitination of expanded polyglutamine protein* in vitro* and may help recruit misfolded proteins as substrates for parkin E3 ubiquitin ligase activity. Therefore, HSP70 can promote the activity of E3 ligase to degrade *α*-synuclein (Figure 6).

Increasing HSPs may also have potential for therapeutic use in PD patients. Several studies suggest that increasing expression of HSP70 reduces alpha synuclein aggregation and toxicity [70–73]. Other HSPs like HSP40 or HSP27 may also reduce *α*-synuclein aggregation [74]. Although one report showed that small molecule inhibitors of HSP90 reduce alpha synuclein oligomer formation and toxicity [75]. Uryu and coworkers demonstrated that expression of HSP90 is increased and associated with alpha-synuclein filaments in the brains of subjects with PD and in a transgenic mouse model of PD, implicating HSP90 in inclusion formation [76].

*Huntington's Disease.* Huntington disease (HD) is a progressive neurodegenerative disorder characterized by abnormal accumulation of huntingtin protein (HTT) due to several repetitions of glutamine (also called polyQ) [77, 78]. Misfolded HTT aggregation causes selective neuronal loss, primarily in the cortex and striatum, and also can lead to cognitive and motor impairments. The repetition of glutamine residue in HTT is critical for its toxicity: that is as the number of CAG repeats increases (CAG: gene code for glutamine), the HTT deposition and neurotoxicity are greater [79–81]. Interestingly, HSP70 is capable of preventing poly-glutamine-induced toxicity in HD models [82]. Increased levels of HSP40, HSP60, HSP70, and HSP100 have been shown to inhibit poly-glutamine-induced protein aggregation and thus attenuate disease progression [82–86]. Experimental data showed that HSP40 and HSP70 prevented intramolecular conformational changes in mutant HTT and attenuated the formation of spherical and annular HTT oligomers, thus promoting the accumulation of less toxic fibrillar and amorphous aggregates [36]. HSP70 has been shown to bind to the HTT exon containing a polyQ expansion primary mouse neurons* in vitro* and in yeast and colocalize to polyQ aggregates* in vitro* and* in vivo*; thus HSP70 might prevent aggregation by binding to a polyQ protein [36, 39]. HSP70 also inhibits oligomerization and fibril formation of polyQ and makes polyQ aggregates more soluble. Several studies reported that both HSP40 and HSP70 inhibit polyQ toxicity in cellular models as well as* in vivo*, and both can inhibit caspase-3 and caspase-9 activity as well as apoptosis-inducing factor (AIF) in HTT-transfected cells [87, 88]. In HD, when the expression level of HSP27 is increased, it prevents poly-glutamine-induced toxicity in neurons [89–91].

**Figure 7:**
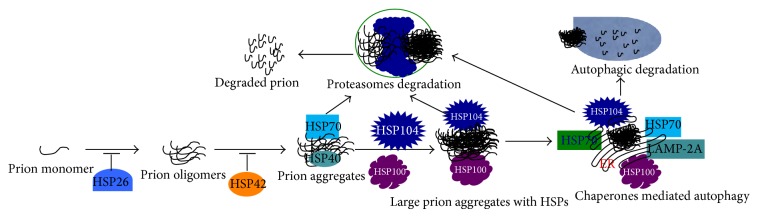
Role of HSPs in prion degradation. Small HSPs such as HSP26 inhibit prion oligomer formation, and HSP42 prevents prionogenesis, whereas HSP70, HSP100, and HSP104 bind with large prion aggregates for degradation through the proteasomal or autophagy pathway.

*Prion Disease.* Prion diseases are a family of rare progressive neurodegenerative disorders that affect both humans and animals. The “prions” are transmitted to tissue and induce abnormal folding of some specific proteins and transform them into pathogenic agents called prion proteins (PrP) [92–96]. Prions can aggregate extracellularly within the CNS to form plaques known as prion plaques, which disrupt neuronal morphology. As a consequence several “holes” are observed in the tissue with resultant spongiform architecture due to vacuole formation in neurons. The central feature of prion diseases is the aggregation of pathologic prion proteins, such as PrP^c^, an abnormal isoform of the cellular prion protein [92–96]. HSP70 binds to aggregated prion proteins and mediates their degradation through the proteasome pathway [36]. HSP70 plays an important role in the propagation of the PrP^c^. Further, in yeast, overexpression of HSP100 leads to disassembly of large prion aggregates and generation of the small prion seeds for new rounds of prion propagation [93] (Figure 7).

Similarly, HSP104 can also inhibit the fibrillation and disassembly of prion peptide* in vitro* [97]. Duennwald et al. reported that yeast sHSPs such as HSP26 and HSP42 could attenuate prionogenesis. With the help of HSP70, HSP42 can prevent conformational rearrangements of prion oligomers, thus attenuating self-assembly, whereas HSP26 can bind to assembled prions oligomers and prevent their aggregation. Furthermore, HSP104, HSP70, and HSP40 destabilize prions and promote their disaggregation [98] (Figure 7).

## 7. Natural Antidotes to Boost the HSP System

Indeed, stimulation of HSPs has emerged as a potential strategy for the treatment of several neurodegenerative diseases [12, 99]. Importantly many nontoxic, naturally occurring small molecules are now known to modulate function of or increase expression of HSPs. Increasing HSP expression in cells often elicits cytoprotective effects [41]. There are several compounds that can increase the heat shock response in cells including the potent HSPs activator, the antibiotic geldanamycin [26, 53, 100]. Compounds that boost the proteasomal pathway to eliminate the misfolded proteins or prevent the misfolding of protein also may have the potential to alleviate neurodegenerative diseases. Recently, naturally occurring traditional medicinal plant-derived, bioactive compounds like curcumin, celastrol, gambogic acid, and withaferin-A have been identified as HSPs activators [101, 102]. The adaptogens extracted from roots of* Eleutherococcus senticosus*,* Schisandra chinensis* berry, and* Rhodiola rosea* are also reported to increase HSP70 when tested in isolated human neuroglia cells [103]. Similarly, ethanolic leave extracts from* Cichorium intybus* and* Jasminum sambac* also induced HSP70 expression in C2C12 myoblasts and rats tissue [104, 105]. All these research reports strongly suggest that natural bioactive phytochemicals have pivotal roles in increasing HSPs expression, which may prevent neurodegenerative diseases.

*Polyphenol Curcumin Is a Natural Inducer of HSPs.* Polyphenol curcumin is derived from the plant* Curcumina longa*. Curcumin has drawn special attention among all the polyphenol compounds because it has several beneficial effects in* in vivo* models of aging, ischemia and trauma [106–110], and animal models of several types of neurodegenerative diseases [31, 108–110]. It is a safe, FDA-approved, and naturally occurring amyloid binding polyphenolic molecule. Its pleiotropic antiamyloid properties suggest that it may have potential for the treatment of several neurodegenerative diseases [111]. Free (native) curcumin (unglucuronidated and unsulfated) readily crosses the blood brain barrier after oral administration and can act as an anti-inflammatory drug, antioxidant, and as an inhibitor of amyloid aggregation [54, 112]. In rodent models of AD, administration of curcumin and related curcuminoids reduced plaque burden and protected against A*β*-toxicity* in vitro* and* in vivo*, thereby improving cognitive function [112–114]. In addition, our finding also suggested that treatment of CAG140 KI mice with 555 parts per million (ppm) of curcumin attenuated neuropathology and transcriptional deficits, including reduction levels of mutant HTT aggregates [29, 114].

Other polyphenols may have similar mechanisms of action, for example, (-) epigallocatechin gallate (EGCG) in green tea [115]. The fact that its binding to aggregates leads to creation of unstructured oligomeric species may be indicative of an alteration in the heat shock response. In fact similar to curcumin, in other cell models, EGCG has been shown that it binds to and inhibits HSP90 by blocking HSP70 association with HSP90 [116, 117]. However opposite to curcumin, it suppresses the induction of HSP70 [118]. Furthermore, our research demonstrated that curcumin administration decreases tau protein aggregation in human tau transgenic (hTauTg) mouse model [32]. Curcumin has also been shown to reduce soluble tau and increase HSPs in a human tau mouse model [26]. These results indicate that even after tangles are established, tau-dependent dysfunction of the synapses and behavior deficits can be corrected by curcumin treatment [26, 32].

Curcumin readily penetrates the brain where it is highly stable [110]. However, it is not stable in plasma and is excessively glucuronidated both in the intestine and by first pass metabolism, which limits penetration into the brain [110, 119–122]. Recently, numerous approaches have been undertaken to improve the bioavailability of curcumin including the use of a glucuronidase inhibitor (e.g., piperine), liposomal curcumin (liposome/micelles), solid lipid curcumin nanoparticles [123], or a curcumin phospholipid complex [124]. Proper formulation is critical to achieve therapeutic levels of curcumin in humans, which show greater glucuronidation than rodents. Feeding mice 500 ppm in chow, a solid lipid nanoparticle containing curcumin (called Longvida, Verdure Sciences [123]), we achieved up to 5 *μ*M of curcumin in mouse brain tissue by two weeks (unpublished observations). Curcumin significantly decreased A*β*-plaque burden and improved memory of the mice model of AD [32, 55, 110]. However, the molecular mechanism(s) behind curcumin's therapeutic effects remain unclear. One possibility is that it is targeting a common endogenous protein clearance pathway, such as the HSP system, that is, dysfunctional in neurodegenerative diseases. To test this hypothesis, we have examined the levels of HSPs and their client proteins (HSP90, HSP70, HSP70, HSP60, HSP40, CDC37, P23, FKBP51, etc.) in HD, tauopathies, and AD animal models of neurodegenerative diseases. We used the CAG140 (140-glutamine codon (CAG)) knockin mouse (CAG140KI) to model HD, which exhibits slowly progressing neurobehavioral impairments and accumulation of large HTT neuropil aggregates [114]. We used the hTau transgenic mouse, which overexpresses wild type human tau with a native promoter [125], and, to model AD, we used the triple transgenic rat (Swedish mutation in APP and mutation in presenilin-1 and presenilin-2) [126]. We observed that there were transgene-dependent reductions in HSPs and their clients, indicating molecular chaperones, are highly affected in all these amyloidogenic models. Interestingly, curcumin delivery was able to restore the levels of HSPs in these transgenic models (unpublished observations). We also looked at the levels of CDC37, P23, and FKBP51. CDC37 inhibited the HSP90 activity, whereas P23 and immunophilin FKBP51 activates it. The HSP90-binding immunophilin FKBP51 is a mitochondrial protein that translocates to the nucleus to protect cells against oxidative stress [127], and its level has been reported to be reduced in AD brain [128]. However, we found that CDC37, P23, and FKBP51 were significantly reduced in all three animal models, suggesting dysregulation of HSP90 cochaperones. In contrast mice fed curcumin showed no reduction in these client proteins.

In contrast, we observed that compared to wild type, there were significant increases in the levels of Fyn (unpublished observations), an important HSP90 client kinase [129], in all three amyloidogenic models, while curcumin reduced Fyn levels, independent from transgene. Dysregulation of Fyn is implicated in tau pathogenesis, because Fyn colocalizes with neurofibrillary tangle in AD brain [129], and it can phosphorylate tau at tyrosine 18 [130]. Further, since HSP90 inhibition selectively degrades many oncogenic client kinases including Src/Fyn [131], this finding further supports that Fyn suppression by curcumin may be via HSP90 inhibition. However, interestingly curcumin did not reduce another HSP90 relevant client protein Akt. The Akt can form complexes with HSP90 and HSP27, and active Akt is stabilized by interaction with HSP90. Recently, the interaction of HSP27 with Akt has been suggested to be involved in regulating apoptosis [132, 133]. Hence, it is possible that HSPs modulate A*β*, tau, and HTT toxicity through interactions with Akt. The transgene dependent loss of HSPs in these animal models may represent a failure to compensate for aggregate HTT, tau, or A*β* oligomers with aging and be relevant to the removal of those toxic aggregates as well as neuroprotection. Moreover, the early synaptic and axonal abnormalities in AD, PD, and HD may be reversed by HSPs [127]. Further, we have also investigated what the minimum dose of curcumin required to modulate HSPs is, and we found that even 0.01 *μ*M of curcumin was able to significantly increase HSP90 and HSP70 after 24 h of incubation in SH-SY5Y cells (unpublished observations). Thus, it is possible that by modulating HSP activity curcumin might inhibit or slow down amyloid formation and eventually reduces neuronal death in different neurodegenerative diseases.

## 8. Conclusion

In summary, protein misfolding and its progressive aggregation inside and outside of the cells are the common features of most of the neurodegenerative diseases. Molecular chaperones such as heat shock proteins play pivotal roles to guard against accumulation of damaged proteins in the cells that could trigger a pathophysiology. Thus harnessing the potential of HSPs is sought for ameliorating the pathology of several neurodegenerative diseases because of its protective role in protein folding and maturation and renaturation of misfolded proteins. Therefore, one of the important strategies to remove the toxic protein aggregates from cells is to boost the endogenous protein clearance pathways by activating the HSPs. Several phytochemicals including curcumin may ameliorate and circumvent deficits in molecular chaperones in amyloidogenic transgenic animal models. Therefore, restoration and upregulation of HSPs by curcumin is a promising therapeutic approach to relieve the adverse effect of accumulated misfolded proteins in several neurodegenerative diseases.

## Figures and Tables

**Table 1:** Common neurodegenerative diseases due to protein misfolding and aggregation. The diseases associated with genetic/risk factors, proteins involved, pathology/diagnostic features, brain areas affected, and clinical symptoms in patients are mentioned below.

Diseases	Genes involved	Risk factors	Proteins involved	Pathology	Affected brain areas	Symptoms
Alzheimer's	APP and presenilin 1, 2	ApoE4	A*β* and Tau	A*β*-plaque and Tau tangle	Hippocampus and frontal cortex	Memory loss, personality change, worried, and depressed

Parkinson's	*α*-Synuclein, Parkin, UCHL-1, and LRRK2	Tau linkage	*α*-Synuclein and tau	Lewy body and tangle	Substantia nigra, striatum, and PFC	Impairment of sensorimotor coordination and cognition

Huntington	Huntingtin (HTT)	Number of CAG repeats in HTT allele	Huntingtin	Inclusion bodies in cytoplasm and nucleus	Striatum	Uncontrolled movements, clumsiness, and balance impairment

Prion	PRNP	Homozygosity at prion codon 129	PrP^Sc^	Prion plaque	Whole CNS	Memory loss, personality change, and movement disorder

Amyotrophic lateral sclerosis	SOD	—	SOD1	Bunina body	Motor neuron of CNS	Disturbances of muscular activity

Multiple sclerosis	HLA, IL2RA, and IL7RA	Kinesin KIF1B, Vit D	—	Demyelinating lesion	White matter of the brain and spinal cord	Physical and cognitive disability

Tauopathies	Tau	Tau-linkage	Tau	Tau tangle	Whole CNS	Memory loss

Lewy bodies dementia	PARK11	E4 allele of ApoE	*α*-Synuclein and ubiquitin	Lewy bodies	Hippocampus, amygdale, and frontal cortex	Impair alertness/attention movement, posture, muscle stiffness, memory loss, hallucinations, and confusion

**Table 2:** Different heat shock proteins, their localization, functions, and involvement in different neurodegenerative diseases.

HSPs	MW (kDa)	Localization	Colocalization	Functions	Involved in diseases
HSP10	10	Mitochondria, cytosol, ER, and nucleus	A*β*	Protein folding	AD, MS, and tauopathies
HSP27	20–30	Cytosol, ER, and nucleus	Tau, A*β*, HTT, and *α*-synuclein	Protein degradation	AD, HD, and PD
HSP40	40	Cytosol	HTT and *α*-synuclein	Protein folding	HD and PD
HSP60	60	Mitochondria	A*β*	Prevent protein aggregation	AD
HSP70	70	Cytosol, ER, nucleus, and mitochondria	A*β*, HTT, *α*-synuclein, and PrP^c^	Protein folding/unfolding	AD, HD, PD, Prion, and MS
HSP90	90	Cytosol and ER	A*β*, HTT, *α*-synuclein, and PrP^c^	Protein degradation and transcription factor	AD, PD, and HD
HSP104/110	100–110	Cytosol and ER	*α*-synuclein and PrP^c^	Thermal tolerance	PD and prion

## Abbreviations

HSP: Heat shock protein
AD: Alzheimer’s disease
PD: Parkinson’s disease
MS: Multiple sclerosis
HD: Huntington’s disease
HTT: Huntingtin
Aβ: Amyloid beta protein
APP: Amyloid precursor protein
PFC: Prefrontal cortex
UCHL1: Ubiquitin carboxy-terminal hydroxylase-L1
${\text{PrP}}^{\text{sc}}$: Prion protein scrapie form
${\text{PrP}}^{\text{c}}$: Prion protein cellular/common
SOD: Superoxide dismutase
CNS: Central nervous system
HLA: Human leukocyte antigen
MW: Molecular weight
ATP: Adenosine triphosphate
ER: Endoplasmic reticulum
sHSPs: Small heat shock proteins
SBS: Substrate binding site
FKBP5: FK506 binding proteins
CYP: Cytochrome P450
TRP: Transient receptor potential
CMA: Chaperone mediated autophagy
LAMP-2A: Lysosome associated membrane protwin-2A
RNAi: Interference ribonucleic acid
HSC70: Heat shock cognate 70
NFT: Neurofibrillary tangle
SNpc: Substantia nigra parts compacta
DA: Dopamine
DAT: Dopamine transporter
AIF: Apoptotic inducing factor
FDA: Food and drug administration
CAG140KI: CAG140 knock in
Tg: Transgenic
APP_Sw_: Amyloid precursor protein Swedish
PS: Presenilin
htauTg: Human tau transgenic
ppm: Parts per million
CHIP: C-terminus of HSC70 interacting protein
CHO: Chinese hamster ovary.
